# Algae-based antioxidant containing selenium yeast (Economase**^®^**) enhanced the growth performance, oxidative stability, and meat quality of broiler chickens

**DOI:** 10.5713/ab.20.0822

**Published:** 2021-04-23

**Authors:** Maleeka N. Nambapana, Samiru S. Wickramasuriya, Shemil Priyan Macelline, K. Samarasinghe, Janak K. Vidanarachchi

**Affiliations:** 1 Department of Animal Science, Faculty of Animal Science and Export Agriculture, Uva Wellassa University, Badulla 90000, Sri Lanka; 2 Department of Animal Science and Biotechnology, Chungnam National University, Daejeon 34134, Korea; 3 School of Life and Environmental Sciences, Faculty of Science, The University of Sydney, Camperdown 2006 NSW, Australia; 4 Department of Animal Science, Faculty of Agriculture, University of Peradeniya, Peradeniya 20400, Sri Lanka

**Keywords:** Algae-based Antioxidant, Broiler, Growth Performance, Meat Quality, Oxidative Stability, Selenium Yeast

## Abstract

**Objective:**

An experiment was conducted to determine the effect of algae-based antioxidant containing Se yeast (EconomasE^®^) on the growth performance, visceral organ weight, meat quality, and oxidative stability of broiler chickens.

**Methods:**

Nine hundred sixty, day-old male broiler chickens (Cobb, 43.97±0.55 g) were divided into three dietary treatments and allocated into 12 deep litter pens in a completely randomized design giving 4 replicate cages for each treatment. Three dietary treatments were: i) control (CON, basal diet with sufficient nutrient), ii) vitamin E (VitE, basal diet supplemented with 100 IU VitE), and iii) Algae-based antioxidant containing Se yeast (EcoE, basal diet supplemented with 0.2% algae-based antioxidant containing Se yeast: EconomasE^®^). Maize soybean meal based basal diets were formulated to meet or exceed the nutrition requirement for broiler chickens. Chickens were fed *ad-libitum* experimental diets during the 42 days experiment period. On days 21 and 42, body weight and feed intake were measured to calculate the feed conversion ratio of the chickens. Intestine and visceral organs were measured together with meat quality and oxidative stability on days 14 and 42.

**Results:**

Chickens fed with EcoE showed improved (p<0.05) growth performance, meat quality, and higher (p<0.05) oxidative stability compared to the chicken fed on CON. Moreover, broiler chickens fed with EcoE showed similar (p<0.05) growth performance with better (p<0.05) meat quality and higher oxidative stability compared to the broiler chickens fed VitE (p<0.05).

**Conclusion:**

The algae-based antioxidant containing Se yeast can be supplemented into commercial broiler diets as a substitution of VitE while maintaining growth performance with enhancing meat quality and oxidative stability of the broiler chickens.

## INTRODUCTION

Reaching the maximum growth potential of broiler chickens in a confined environment within the short life span usually causes huge stress to the chickens. During the rearing period, broilers may experience different types of stress such as environmental stress including heat stress and stocking density, nutritional stress, technological and management-related stress [1]. Under this kind of stress, excess free radicals are generated and compromised the antioxidant defense system of the chickens resulting in oxidative stress. The weak antioxidant defense system with the inability to maintain optimal redox balance leads to abnormal oxidative stress causing cell components damaged, lipid peroxidation, DNA damage, and weaken the immune system of broiler chickens [2]. A compromised immune system will result in increased morbidity and mortality rates and decrease broiler production efficiency [3]. Besides, increases oxidative damage which adversely affects body weight (BW) gain, feed consumption, carcass weight, and meat quality of broiler chicken [4].

As a tool to manage serious economic consequences commensurate with various pathological and welfare issues, lowering oxidative stress is a key interest in broiler production [5]. To avoid oxidative stress in broilers, different dietary antioxidants were tested for chickens [6,7]. Among those dietary strategies, vitamins such as ascorbic acid and α-tocopherol, minerals like zinc and selenium, and polyphenol compounds with antioxidant properties are incorporated in broiler diets to reduce oxidative damages in chickens [8]. Possible application of selenium and selenoproteins as antioxidants to mitigate oxidative stress during intestinal inflammation also reported [9]. Selenoproteins such as glutathione peroxidase 2, decreased the activation of nuclear factor kappa B and thereby reduced the oxidative stress [9]. Moreover, series of selenium studies conducted by Lee and colleagues [10,11] reported that sodium selenite, selenium-enriched yeast, and B-taxim selenium significantly reduced the antioxidant-related enzyme activities in the chicken with the enteric challenge models.

There has been increasing concern on commercially producing value-added seaweed-based feed additives for broilers with its huge potential for reducing oxidative stress and improving growth performance. An algae-based antioxidant supplement containing selenium yeast (Se yeast) is one such product that showed a beneficial impact on oxidative stress, growth performance, and meat quality attributes in broiler chickens [12]. According to Xiao et al [13], algae-based antioxidants containing Se yeast (Economase^®^ E; Alltech Inc., Nicholasville, KY, USA) replace the vitamin E (VitE) requirements by maintaining growth performance, meat quality, and health status of the chickens. Similarly, Nambapana et al [14] reported Economase^®^ E enhanced or maintained the growth performance and meat quality of the chickens. However, there is less literature available on an algae-based antioxidant supplement containing Se yeast and its effect on broiler chickens under actual farming conditions. Therefore, this study was conducted to determine the effect of algae-based antioxidant containing Se yeast (EconomasE^®^) on the growth performance, visceral organ metrics, meat quality, and oxidative stability of broiler chickens reared under commercial farming conditions.

## MATERIALS AND METHODS

### Experimental design, management, and animal care

Nine hundred sixty (960), day-old male broiler chicks (Cobb) vaccinated against Marek’s disease, Infectious bronchitis, and Newcastle disease were obtained from a local hatchery (Nelna Breeders [Pvt] Ltd, Embilipitiya, Sri Lanka). On arrival, birds were weighed (43.97±0.55 g) and allocated into one of three treatments in a completely randomized design. Each treatment consisted of four replicate pens with 80 chickens per pen. Three dietary treatments were: i) control (CON, basal diet with sufficient nutrient), ii) VitE (basal diet supplemented with 100 IU VitE), iii) algae-based antioxidant (EcoE: basal diet supplemented with 0.2% algae-based antioxidant containing Se yeast).

Twelve deep litter pens (9.8 m^2^/pen) littered with rice husks were used in this experiment in an open-sided house system. Chickens were allowed *ad libitum* access to experimental diets and fresh clean water via equipped round feeders and bell drinkers throughout the experiment. Floor brooding was performed for 14 days period with 24 hours lighting schedule. The temperature was maintained 34°C during the brooding period (67%±3% relative humidity) and gradually acclimatized to environment temperature (28°C). The experiment lasted for 42 days and chickens were observed twice a day for general conditions. All the management practices were conducted according to the guidelines provided by the Cobb broiler management guide [15]. All the experimental procedures were followed by the specific guidelines for ethical considerations presented in the Guide for the Care and Use of Agricultural Animals in Research and Teaching, 3rd edition [16].

### Experimental diets

Maize soybean meal based basal diets were formulated (Table 1) to meet or exceed the nutrition requirement for broiler chickens [17]. The dietary treatments were produced by supplementing 100 IU/kg VitE and Algae-based antioxidant containing Se yeast (EconomasE; Alltech Inc., USA) into basal diets. All the experimental diets were fed in a mash form.

### Growth measurements

Pen-based BW and feed intake (FI) were measured on days 21 and 42. Average daily gain (ADG) and average daily feed intake (ADFI) were calculated by dividing the number of birds in each replicate pen. Mortalities were recorded daily when occurred. Mortality corrected feed conversion ratio (FCR) were calculated using ADG and ADFI.

### Post-mortem and sample collection

Experimental diet samples were collected at the end of the diet mixing and stored for nutrient analysis. On day 14, five chickens from each replicate pen were randomly selected, recorded the live BW, and euthanized humanely by cervical dislocation for sample collections. Visceral organs (liver, heart, spleen, bursa, gizzard, pancreas, and ceca) and small intestine (duodenum, jejunum, and ileum; as described [18] were excised separately. Remain contents in the organ were removed manually, blotted dry, and recorded the weights.

At the end of the study on day 42, five chickens from each pen were selected randomly and starved for 12 hours before sacrifice. Live BW were recorded and sacrificed the chickens by cervical dislocation followed by bleeding for three minutes. Birds were mechanically scalded at 55°C and de-feathered. Visceral organs and small intestine were removed manually, blotted dry, and weighted similar to day 14. Afterward, breast muscles including pectoralis major and pectoralis minor were removed, snap-frozen, and stored at −20°C for further analysis.

### Sample preparation and laboratory analysis

#### Nutrient analysis of feed

Composite samples of experimental feeds were taken and prepared for analysis. Crude protein and gross energy analysis were performed according to the methodologies of AOAC [19].

#### Meat quality analysis

Ultimate pH, color, drip loss, cook loss, water holding capacity (WHC), oxidative stability (thiobarbituric acid reactive substances [TBARS]), and tenderness (shear force value) were measured as previously described [20].

#### Determination of selenium content in breast meat

Selenium content of fresh broiler breast meat was determined according to AOAC [19] methodology. Chicken breast meat samples were obtained from the same place at each breast portion and dried for 48 hours at 120°C to remove the moisture. From each dried sample, 0.3 g of meat sample was transferred into a decontaminated decomposition vessel and 5 mL HNO_3_ was added to the vessel. The closed vessel was heated in a drying oven (IncuMax CV150, 3951C Industrial Way, Concord, CA, USA) at 150°C for 2 hours, and then the content was cooled to room temperature and transferred to a 10 mL volumetric flask. Distilled water (4 mL) was added to the volumetric flask and mixed well and the content was top up with the distilled water. An aliquot digested test solution was pipetted into a decontaminated 50 mL round, flat–bottom borosilicate flask, and 1 mL Mg (NO_3_) solution (2 M) was added and heated on a hot plate at low heat to dryness. The flask was placed in a furnace at 450°C to oxidize carbonaceous compounds and excess Mg (NO_3_) was decomposed. The content was cooled to room temperature and 2 mL 8 N HCl was added to the flask and covered with a watch glass and the flask was placed on the steam bath for 10 minutes and the content was then cooled to room temperature.

The selenium stock solution was prepared by dissolving 1 g Se powder in minimum volume HNO_3_ in a 200 mL beaker and evaporated to dryness. Then, 2 mL H_2_O was added and evaporated again to dryness and repeated twice and the concentration of the stock solution was 1 mg/mL. The residue was dissolved in minimum volume HCl (1+9) in a 1 L volumetric flask and diluted with HCl. The working solutions were prepared as 1, 2, 3, 4, 5, 6 μg/mL. Stock solution was transferred (10 mL) into a 100 mL volumetric flask and the content was diluted with distilled water. 1, 2, 3, 4, 5, 6 mL of diluted solution were transferred into separate 100 mL volumetric flasks and diluted with distilled water.

Calibration curve of μg selenium against absorption (196 nm) was obtained by using selenium lamp (in place and recorder set for 20 mm/min) and standards and μg Selenium in sample aliquots were obtained from the same curve.

### Calculation and statistical analysis

Relative weights of organs and relative lengths of small intestine segments were calculated according to the following formula before the statistical analysis;


$$\begin{array}{l} Relative\;weight\;of\;the\;organ=\frac{fresh\;organ\;weight(g)}{live\;weight\;of\;brid(g)}\times 100\\ Relative\;length\;of\;small\;intestine\;segment=\frac{fresh\;segment\;length\;(cm)}{live\;weight\;of\;brid(g)}\times 100 \end{array}$$

Each replicate pen was considered as the experimental unit for growth performance. Obtained data from the selected individual chickens for visceral organ and intestinal weight were pooled to get the pen-based mean before data analysis. Then, data were analyzed using the general linear model of the One-way analysis of variance procedure of SAS software (SAS Inst. Inc., Cary, NC, USA). Statistical significance (p< 0.05) of differences among treatment means was assessed using Duncan’s multiple range test.


$$Y_{ijk}=\mu +T_{i}+R_{j}+e_{ijk}$$

Where, *Y_ijk_* = response variable, μ = sample mean, *T_i_* = effect of ith *T* (0, 1, and *T* 2 [BW, FI, FCR, thiobarbituric acid, L*, a*, b*, drip loss, cook loss, shear force, WHC, pH]), *R_j_* = effect of jth replicate (1, 2, 3, and 4), *e_ijk_* = experimental error.

## RESULTS

### Growth performance

The effect of dietary algae-based antioxidant supplementation on the growth performance of the broiler chickens from hatch to 42 days of age is presented in Table 2. Broiler chickens fed EcoE were shown higher BW (p<0.05) compared to chickens fed CON diet on days 21 and 42. Moreover, chickens fed EcoE were heavier (p<0.05) than the chicken fed VitE on day 21. Nevertheless, no BW deference was observed among chickens fed VitE and EcoE when they reached day 42. Following the BW data, the chickens fed the diet with EcoE had higher ADG (p<0.05) compared to CON during the grower period (22 to 42 d) and overall period (1 to 24 d).

Chickens fed the CON diet consumed more feed (p<0.05) compared to EcoE fed chickens during the grower period (22 to 42 d) and overall period (1 to 24 d). However, no FI deference was observed among VitE and EcoE fed chickens during the 42 days study period.

Similar to BW and ADG data, chickens fed the EcoE diet were shown an improved (p<0.05) FCR compared to chickens fed the CON diet during the starter period (1 to 21 d), grower period (22 to 42 d), and overall period (1 to 24 d). Chickens fed VitE showed a similar (p>0.05) FCR compared to chickens fed the CON diet during the starter period (1 to 21 d) and overall period (1 to 24 d). Nonetheless, improved FCR (p<0.05) was observed from the VitE fed chickens compared to those fed the CON diet during the grower period (22 to 42 d) and it was similar (p>0.05) to EcoE fed chickens.

### Visceral organ weights

Dietary treatment effects on visceral organ weights of the broiler chickens are shown in Table 3. Chickens fed the EcoE diet were shown higher (p<0.05) gizzard, spleen, and bursa weight compared to chickens fed the CON diet on days 14 and 42. Similarly, higher (p<0.05) bursa weight was observed in the chickens fed with VitE diet compared to chickens fed with CON diet on days 14 and 42. However, bursa weight was comparatively higher (p<0.05) in the chickens fed EcoE diet compared to the chickens fed VitE diet on days 14 and 42. Higher (p<0.05) liver and caeca weight were observed in chickens fed VitE diet compared to the chickens fed EcoE diet on days 14 and 42.

### Small intestine measurements

The relative weight and length of the intestine of the birds fed treatment diets are shown in Table 4. Birds fed with VitE and EcoE were shown heavier (p<0.05) duodenum and ileum weight compared to the birds fed CON diet on days 14 and 42. Nevertheless, there were no duodenum and ileum weight differences (p>0.05) were observed between VitE and EcoE fed birds on day 14 and 42. However, jejunum weight was higher (p<0.05) in EcoE fed birds compared to the birds fed with VitE on days 14 and 42.

Longer duodenum, jejunum, and ileum length (p<0.05) were observed from the birds fed VitE diet compared to the birds fed CON diets on day 14. Meantime, on day 14, birds fed EcoE were shown similar (p>0.05) duodenum and jejunum length, and higher (p<0.05) ileum length compared to the birds fed CON diets. On day 42, higher duodenum length (p<0.05) and lower ileum length (p<0.05) were observed in the birds fed EcoE diet compared to the birds fed with CON diet.

### Meat quality

Meat quality parameters of the broiler chickens fed with three different dietary treatments on day 42 are shown in Table 5. Lower lightness (L*) and yellowness (b*) together with higher redness (a*) (p<0.05) were observed in meat from the EcoE fed birds compared to the CON and VitE diets. Birds fed the VitE diet were shown higher (p<0.05) lightness (L*) and yellowness (b*) in the meat compared to the birds fed EcoE. Higher pH, WHC, and shear force (p<0.05) were observed in the meat from EcoE fed chickens compared to those fed the CON diet. Moreover, meat from EcoE fed birds were shown lower (p<0.05) drip loss and cooking loss compared to the meat from CON birds. Meat from the birds fed VitE diet was shown higher (p<0.05) drip loss and cooking loss commensurate with lower WHC compared to the meat of birds fed EcoE diet.

Showing the oxidative stress status of the birds, a lower TBARS value (p<0.05) was observed in the breast meat from birds fed EcoE diet compared to the CON birds. Moreover, the TBARS value of the EcoE fed chickens were lowered (p<0.05) compared to the chickens fed VitE diets. Selenium content was higher (p<0.05) in the bird fed EcoE diet compared to CON and VitE counterparts. No difference (p>0.05) was observed for Se content in breast muscle from chickens fed CON and VitE.

## DISCUSSION

Oxidative stress in broiler production became a hot topic for researchers as it occurs in chickens become commonplace. Higher growth potentials, heat stress, and low-quality feed materials are the reasons for the oxidative stress in chickens. Mitigating measures, such as antioxidant feed additives are implementing in commercial broiler productions to enhance or maintain the optimum growth performance and meat quality.

Among these antioxidant feed additives, VitE plays a major role in animal feed and has been credited to improve the anti-oxidative status of animals [13]. Additionally, VitE is involved in other metabolic processes, cell signaling, and immune functions [13]. However, increase the VitE cost limits its maximum incorporation into the feed and leads to the search for cost-effective VitE replacers with better antioxidant properties. Some VitE replacers, such as polyphenols from grapes, blackberries, citrus, and sweet chestnuts were tested for chickens [21]. Further, algae-based antioxidant containing Se yeast has been reported as a potential VitE replacer for commercial broilers [14,22]. Nevertheless, fewer studies were conducted to evaluate the effects of algae-based antioxidant containing Se yeast on broiler performance, oxidative stability, and meat quality under commercial farming conditions. With this persuasion, this study tested the hypothesis that algae-based antioxidant containing Se yeast (EconomasE^®^) would improve the growth performance, meat quality, and oxidative stability of broiler chickens reared under commercial farming conditions.

According to the interesting meta-analysis conducted by Pompeu et al [23] using 51 scientific publications, the addition of VitE did not affect significantly on growth performance of broiler chickens. This is in agreement with the present study that confirmed the growth performance of broiler chickens was not affected by the addition of VitE into broiler diets. This confirmed the fact that VitE availability from primary diet ingredients such as corn and soybean meal is sufficient to ensure the optimum growth performance of the chicken under normal farming conditions. Nevertheless, improved growth performance with VitE together with Se was reported in broiler chickens under heat stress conditions [24]. Antioxidant properties of VitE that supports to maintain the redox balance and thereby reduce oxidative stress may attribute to the improved growth performance during the heat stress. Even though VitE did not influence growth performance, algae-based antioxidant containing Se yeast (EconomasE^®^) as a replacement of VitE was shown an improved or maintain the growth performance of broiler chickens [13,14,25]. With the agreement, improved feed efficiency commensurate with lower FI and higher weight gain were observed in broiler chickens fed EcoE in this study. According to the breast muscle gene expression profile study [13], EcoE greatly mimics the VitE gene expression profiles for skeletal and muscular system development and function. This providing clues of the reason for the improved growth performance of broiler chickens fed EcoE and VitE in this study. Moreover, significant growth improvement in EcoE fed birds compared to VitE fed birds may be ascribed to the fact that EcoE contained Se and other micronutrients from algae that may have the ability to trigger antioxidant system and unknown gene expression for growth improvement. With solid evidence, previous studies [26] were reported that the diet supplemented with microalgae enhanced the growth performance of broiler chickens.

Selenium is known as one of the essential micronutrients in poultry required for normal growth and maintenance [27]. Moreover, Se is important for terminating free radicals in animal body tissues by enhancing the function of glutathione peroxidase enzymes and to protect poultry from exudative diathesis and pancreatic fibrosis [28]. The recommended Se level in broiler diets was set as 0.15 g/kg [17]; besides, this level often can be fulfilled by the feedstuffs in the diets. However, the maximum level of Se supplementation of 0.3 ppm recommended by the United States Food and Drug Administration. High Se content in the fish meal was reported by Arthur [29] for instance Se concentration in fishmeal is 1.96 ppm (14 samples) while soybean meal contains 0.12 ppm (32 samples) and corn has 0.04 ppm (45 samples). Therefore, a high inclusion level of fishmeal in the starter diet may result in a higher Se level than the finisher diet (48.5 vs 20.0 g/kg), and supplemented Se sources like EcoE may have more impact in the finisher phase than starter phase. Interestingly, the positive impact of EcoE is prominent in the finisher phase than starter phase in the present study such that EcoE improved the weight gain by 3.12% and 10.4% than CON diet in starter and finisher phase respectively. Likewise, in starter and finisher phases, EcoE improved the FCR by 6.97% and 16.8% than CON, respectively. Further, EcoE consisted of dried *Aspergillus niger* fermentation extract which includes more effective lipases, proteases, pectinases, cellulases, hemicellulases, and xylanases [30]. These enzymes could degrade tannins associated with cell wall polysaccharides. Consequently, these enzymes may affect the FCR of broilers. Thus, it is reasonable to hypothesis that EcoE treatment might have contributed to better nutrient utilization and improved the growth performance of broilers.

As the largest immunological organ, robust intestine and associated visceral organs will make a healthier animal with efficient digestion and utilization of nutrients [31]. For the comprehensive understanding, visceral organ weight and small intestine weights together with lengths were measured in this study. Supplementation of VitE and EcoE was shown to increase the visceral organ weights and intestinal measurements compared to CON fed chickens. Similar to our results, Nambapana et al [14] observed a higher relative weight of bursa, spleen, and caeca in chickens fed VitE and EcoE on day 14. Moreover, higher gizzard, spleen, and bursa relative weights were reported in chickens fed VitE and EcoE on day 42 [14]. Likewise, supplementation of green algae (*Chlorella vulgaris*) and red algae powder (*Chondrus crispus*) was increased the relative weight of the lymphoid organs such as bursa and thymus of the broiler chickens [32,33]. According to the literature, a higher size of lymphoid organ means the better immunological activity and health status of the chickens [33]. Hence, increased lymphoid organ weights in the present study may attribute to the activation and development of immunity, T-cells, and thymus function of the broiler chickens fed with EcoE. Similar to lymphoid organs, birds fed with the EcoE diet was shown higher gizzard weight on days 14 and 42. Nambapana et al [14], also reported that EcoE addition improved the gizzard weight of the chickens on day 42. Increasing gizzard may allow for better mechanical digestion of feed and make more nutrients available for chickens [34]. Additionally, observed higher visceral organ weight in chicken fed with algae-based antioxidant containing Se yeast in this study may follow the notion raised by Mohamed et al [35]. According to Mohamed et al [35], organic forms of selenium such as Se yeast have better bioavailability and proteins (selenocysteine and selenomethionine) that can be retained efficiently in the body tissues. Observed higher intestinal weights and length in the bird fed EcoE diet in this study manifest the fact that it allows better nutrient digestion and absorption followed by improved growth performances.

In the broiler chickens, dietary supplementation of VitE has been extensively studied for enhanced antioxidant capacity and thereby improved meat quality [13]. Similarly, algae and algae-based products have been tested to improve meat quality and reduce oxidative stress in broiler chickens [33]. According to the previous study conducted with algae-based antioxidant containing Se yeast, supplementation of algae-based antioxidant containing Se yeast into broiler diet enhance the meat quality and oxidative stability of the chickens [14,22]. With the same conviction, we performed meat quality and oxidation stability analysis in this study. Our observed results ensured the fact that supplementation of algae-based antioxidant containing Se yeast into broiler diet enhanced the meat quality and oxidation stability. Especially, the observed lower TBARS value in EcoE fed chickens confirmed the notion that Se (in its organic form) optimized the action of glutathione peroxidase activity and thereby lowered the free radical surge in the tissues [36].

As claimed by previous studies, it concluded that the retention of selenium in chicken breast muscle is accompanied by the selenium and the vit E content in the chicken diet [24]. Similarly, Choct et al [37] and Ševčíková et al [27] reported supplementation of Seyeast into broiler diets increased the selenium retention in the chicken breast muscle. Further, Surai [1] reported that selenomethionine was actively absorbed in the intestine by a similar process as absorption of methionine while retaining in all tissues as protein. With the agreement of those observed results, supplementation of algae-based antioxidant containing Se yeast into broiler diet showed increased selenium retention in the breast muscles of the present study. Hence, feeding chicken with EcoE supplemented diet could be an effective way to produce Se enriched meat and thereby increased the market value.

Overall, it can be concluded that supplementation of algae-based antioxidant containing Se yeast at the rate of 0.2% improved growth performance, meat quality, and oxidative stability of the commercial broiler chickens from hatch to 42 days.

## Figures and Tables

**Table 1:** Composition of the basal diets (%, as fed basis)

Items	Broiler starter (1 to 21 d)	Broiler finisher (22 to 42 d)
Ingredients (%)		
Maize	49.00	40.00
Soybean meal 44%	27.70	19.00
Rice polish	8.28	21.75
Fish meal 60%	4.85	2.00
Coconut poonac	5.00	10.00
Shell grit powder	1.08	2.00
Dicalcium phosphate	0.50	0.40
Lysine HCl	0.10	0.50
DL-methionine	0.20	0.40
Coconut oil	2.00	3.40
Salt	0.25	0.25
Coccidiostat^1)^	0.02	-
Vitamin mineral premix^2)^	0.30	0.30
Nutrient composition^3)^		
Metabolizable energy (kcal/kg)	3,033	3,177
Crude protein (%)	23.60	19.92
Lysine (%)	1.29	1.12
Methionine (%)	0.53	0.55
Methionine+cysteine (%)	0.97	0.89
Available phosphorus (%)	0.46	0.38
Analyzed value^4)^		
Gross energy (kcal/kg)	4,132	4,299
Crude protein (%)	24.3	21.1

1) COCCIVAC^®^-D2, Merck Animal Health.

2) 1 kg of vitamin mineral premix contained 1,000,000 IU vitamin A, 150,000 IU vitamin D_3_, 800.0 mg vitamin B_1_, 100.0 mg vitamin B_2_, 30.0 mg vitamin B_3_, 20.0 mg vitamin B_6_, 0.5 mg vitamin B_12_, 2,500.0 mg vitamin E, 10,000 mg vitamin C, 23% calcium, 20.0 mg vitamin K_3_, 160.0 mg niacin, 4,000.0 mg cholin, 1.5% DL-methione, 8% phosphorous, 4% magnesium, 1% sodium, 0.5% lysine, 1,500.0 mg iron (sulfate), 100.0 mg copper (sulfate), 4,000.0 mg zinc (oxide), 100.0 mg iodine (potassium), 50.0 mg and cobalt (carbonate).

3) The values were calculated according to the values of feedstuffs in NRC (161994).

4) Data are the mean of triplicate analyses of each diet sample.

**Table 2:** Effect of EconomasE^®^ on the growth performance of the broiler chickens (mean±standard error)

Item	Treatment^1)^			p-value

	CON	VitE	EcoE	
Body weight (g)				
Day 21	669±1.53^a^	665±1.48^a^	697±2.27^b^	0.002
Day 42	2,136±4.08^a^	2,204±5.39^ab^	2,290±3.74^b^	0.025
ADG (g/bird/d)				
1 to 21 d	32±0.26	32±0.32	33±0.43	0.060
22 to 42 d	48±0.72^a^	50±0.66^a^	53±0.22^b^	0.002
1 to 42 d	50±0.97^a^	51±1.02^ab^	54±1.02^b^	0.001
ADFI (g/bird/d)				
1 to 21 d	59±9.14	58±9.52	57±9.66	0.057
22 to 42 d	140±9.63^b^	137±8.44^ab^	132±8.60^a^	0.001
1 to 42 d	97±6.92^b^	96±6.53^ab^	95±5.14^a^	0.001
FCR (g/g)				
1 to 21 d	1.84±0.22^b^	1.81±0.43^b^	1.72±0.11^a^	0.002
22 to 42 d	2.91±0.12^b^	2.74±0.51^a^	2.49±0.31^a^	0.040
1 to 42 d	1.87±0.21^b^	1.84±0.22^ab^	1.76±0.12^a^	0.001

ADG, average daily gain; ADFI, average daily feed intake; FCR, feed conversion ratio.

1) CON, basal diet with sufficient nutrient; VitE, basal diet supplemented with 100 IU vitamin E; EcoE, basal diet supplemented with 0.2% algae-based antioxidant supplement.

a,b Means with different superscripts within the same row are significantly different (p<0.05).

**Table 3:** Effect of EconomasE^®^ on the visceral organ weight1) of the broiler chickens (mean±standard error)

Item	Treatment^2)^			p-value

	CON	VitE	EcoE	
Day 14				
Proventriculus	0.70±0.01^a^	0.76±0.02^b^	0.69±0.01^a^	0.050
Gizzard	2.79±0.09^a^	2.99±0.08^ab^	3.13±0.06^b^	0.004
Pancrease	0.51±0.02^ab^	0.52±0.02^b^	0.46±0.02^a^	0.033
Liver	3.01±0.03^a^	3.11±0.03^b^	3.01±0.02^a^	0.006
Heart	7.30±0.01	7.50±0.01	7.50±0.01	0.230
Spleen	0.09±0.002^a^	0.11±0.002^b^	0.11±0.001^b^	0.001
Bursa	0.26±0.004^a^	0.32±0.007^b^	0.39±0.004^c^	0.001
Caeca	0.95±0.05^a^	1.13±0.06^b^	1.02±0.04^a^	0.008
Day 42				
Proventriculus	0.41±0.01	0.40±0.01	0.41±0.01	0.321
Gizzard	1.38±0.03^a^	1.51±0.03^b^	1.45±0.05^b^	0.010
Pancrease	0.19±0.01^a^	0.22±0.01^b^	0.21±0.01^ab^	0.006
Liver	1.71±0.02^b^	1.70±0.04^b^	1.64±0.04^a^	0.024
Heart	0.42±0.01^a^	0.41±0.01^b^	0.42±0.01^a^	0.005
Spleen	0.10±0.01^a^	0.11±0.01^ab^	0.13±0.01^b^	0.001
Bursa	0.25±0.01^a^	0.27±0.01^b^	0.30±0.01^c^	0.001
Caeca	0.45±0.01^b^	0.64±0.02^a^	0.45±0.02^b^	0.023

1) Organ weights were express as a proportions of live body weight.

2) CON, basal diet with sufficient nutrient; VitE, basal diet supplemented with 100 IU vitamin E; EcoE, basal diet supplemented with 0.2% algae-based antioxidant supplement.

a–c Means with different superscripts within the same row are significantly different (p<0.05).

**Table 4:** Effect of EconomasE^®^ on the relative weight and the length1) of the small intestine segments of broiler chickens (mean±standard error)

Item	Treatment^2)^			p-value

	CON	VitE	EcoE	
Day 14				
Duodenum weight	0.97±0.01^a^	1.05±0.01^b^	1.04±0.01^b^	0.001
Jejunum weight	1.29±0.02^a^	1.62±0.02^b^	1.78±0.03^c^	0.001
Ileum weight	1.04±0.02^a^	1.12±0.02^b^	1.09±0.01^b^	0.001
Duodenum length	6.25±0.05^a^	6.47±0.04^b^	6.22±0.02^a^	0.050
Jejunum length	1.37±0.11^a^	1.40±0.11^b^	1.39±0.11^ab^	0.023
Ileum length	7.93±0.07^a^	8.55±0.08^c^	8.23±0.12^b^	0.010
Day 42				
Duodenum weight	0.43±0.02^a^	0.52±0.02^b^	0.49±0.02^b^	0.001
Jejunum weight	1.06±0.04^a^	1.08±0.03^a^	1.11±0.04^b^	0.001
Ileum weight	0.53±0.04^a^	0.59±0.03^b^	0.64±0.04^b^	0.001
Duodenum length	1.71±0.03^a^	1.72±0.04^a^	1.65±0.02^b^	0.043
Jejunum length	3.96±0.04^b^	3.92±0.04^a^	4.01±0.05^b^	0.006
Ileum length	4.03±0.03^b^	3.97±0.02^a^	3.88±0.03^a^	0.010

1) Relative weight and lengths were express as a proportions of live body weight.

2) CON, basal diet with sufficient nutrient; VitE, basal diet supplemented with 100 IU vitamin E; EcoE, basal diet supplemented with 0.2% algae-based antioxidant supplement.

a–c Means with different superscripts within the same row are significantly different (p<0.05).

**Table 5:** Effect of EconomasE^®^ on the meat quality parameters of the broiler chickens on day 42 (mean± standard error)

Item	Treatment^1)^			p-value

	CON	VitE	EcoE	
Meat color				
Lightness (L*)	57.10±0.26^b^	56.70±0.23^b^	54.30±0.24^a^	0.001
Redness (a*)	2.10±0.04^a^	2.20±0.04^b^	2.30±0.05^c^	0.001
Yellowness (b*)	14.80±0.06^c^	14.40±0.06^b^	13.50±0.08^a^	0.001
pH	5.70±0.04	5.80±0.06	5.80±0.03	0.055
Drip loss %	6.10±0.12^c^	5.70±0.11^b^	3.90±0.08^a^	0.001
Cooking loss %	19.30±0.17^c^	17.50±0.32^b^	13.30±0.14^a^	0.001
WHC %	48.90±0.67^a^	52.50±0.35^b^	54.50±0.25^c^	0.001
Shear force (N)	27.40±1.47^a^	32.70±1.69^b^	33.50±1.09^b^	0.001
TBARS (mg/kg)	0.26±0.01^c^	0.22±0.01^b^	0.19±0.01^a^	0.001
Selenium content (mg/L)	3.90±0.15^a^	4.30±0.19^a^	5.20±0.17^b^	0.001

WHC, water holding capacity; TBARS, thiobarbituric acid reactive substances.

1) CON, basal diet with sufficient nutrient; VitE, basal diet supplemented with 100 IU vitamin E; EcoE, basal diet supplemented with 0.2% algae-based antioxidant supplement.

a–c Means with different superscripts within the same row are significantly different (p<0.05).
